# The association of manganese levels with red cell distribution width: A population-based study

**DOI:** 10.1371/journal.pone.0292569

**Published:** 2024-08-15

**Authors:** Guanmian Dai, Huanhuan Sun, Yanli Lan, Jinhong Jiang, Bingmu Fang

**Affiliations:** 1 Department of Hematology, Lishui People’s Hospital, Lishui, Zhejiang, China; 2 Department of Traditional Chinese Medicine, FuYang Women and Children’s Hospital, Fuyang, Anhui, China; 3 Department of Oncology, Lishui People’s Hospital, Lishui, Zhejiang, China; University of Cape Town Faculty of Science, SOUTH AFRICA

## Abstract

**Objectives:**

Experimental and acute exposure studies imply that manganese affects red blood cell production. Nevertheless, the association between environmental exposure and red blood cell distribution width (RDW) has yet to be explored. This research sought to assess the correlation between blood manganese levels and RDW within the general population of the United States.

**Materials and methods:**

Employing weighted multiple linear regression models, data from the 2011–2018 National Health and Nutrition Examination Survey (NHANES) were utilized to assess the correlation between manganese levels in the blood and RDW. Restricted cubic spline plots and two-piecewise linear regression models were also employed.

**Result:**

The analysis included a total of 15882 participants in which we determined an independent positive relationship between blood manganese levels and RDW among participants(β = 0.079, *P*<0.001). Moreover, we identified a J-shaped association between blood manganese levels and RDW in total participants (inflection point for blood manganese: 7.32 ug/L) and distinct subgroups following adjusted covariates. Women exhibited a more pronounced association, even after controlling for adjusted covariates.

**Conclusions:**

We determined a J-shaped relationship between blood manganese levels and RDW with an inflection point at 7.32 ug/L for blood manganese. Nevertheless, fundamental research and large sample prospective studies are needed to determine the extent to which blood manganese levels correlate with RDW.

## Introduction

Manganese is a metallic element crucial for numerous physiological and developmental functions, including body growth, energy homeostasis, immunity, oxygen metabolism, and the formation of bone [1, 2]. Despite the fact that it is vital in trace amounts, manganese overexposure can elicit developmental toxicity, oxidative stress, as well as inflammatory response in embryos [3, 4]. Epidemiologic research has revealed that an inverse U-shaped curve characterizes the relation between manganese and birth weight [5, 6]. In recent times, the contamination of drinking water, gasoline additives, and agricultural fungicides have emerged as anthropogenic sources that have significantly augmented the burden of excessive environmental manganese [7, 8]. Despite the fact that occupational exposure appears in specific industrial processes such as smelting operations and welding, the general population also encounters the danger of manganese exposure. Diet is the principal route of manganese consumption in humans, and adequate daily intake levels for this metal vary according to factors such as age, gender, and nutritional status. These variables impact absorption rates, which can vary between 1% and 5% [9–11]. The inhalation of manganese is concerning because of its excellent absorption rates, particularly within the brain [12, 13]. Previous studies found that excessive manganese in the human body could result in nervous system complications such as Parkinsonism, Alzheimer’s, Huntington’s, and even damage to liver and heart [14–17]. Recently, concern among researchers regarding the role of blood manganese levels in a variety of diseases has grown. Consequently, it is significant to evaluate the association between blood manganese levels and related blood indexes.

The red blood cell distribution width (RDW) is routinely provided as part of a complete blood count (CBC). A normal RDW implies homogeneous red-blood-cell(RBC) size, whereas an elevated RDW indicates heterogeneous RBC size, recognized as anisocytosis. The occurrence of an RDW value falling below the standard reference value is rare and lacks clinical significance. An elevated RDW frequently represents the states of chronic systemic inflammation, malnutrition, and microcirculatory disorders [18, 19]. Recent studies have demonstrated that elevated RDW values are substantially associated with the prognosis in patients with numerous diseases, such as sepsis [20], atrial fibrillation [21], immune diseases [22], as well as other chronic health conditions [23]. Notably, Peters et al. recently demonstrated that elevated RDW values were associated with exposure to heavy metals, including cadmium and lead [24]. Nevertheless, the correlation between blood manganese levels and RDW in the general population has only been investigated in a limited number of studies. RDW, as an inflammatory marker, has been prevalently used in evaluating the severity and prognosis of various diseases due to the fact that it is noninvasive, economical and convenient [25–27]. National Health and Nutrition Examination Survey (NHANES) is a research program designed to collect detailed information concerning the health and nutrition of the United States population. Nonetheless, to date, there has been no exploration by researchers into the correlation between blood manganese levels and RDW by analyzing data obtained from NHANES. Thus, our aim in the present study was to explore the relationship between blood manganese levels and RDW on the basis of a representative population sample from NHANES. We conjectured that elevated RDW might be associated with increased blood manganese levels.

## Materials and methods

### Study design and participants

The National Health and Nutrition Examination Survey (NHANES) is a population-based cross-sectional survey designed to assess the health and nutritional status of the population in the United States. The NHANES interview includes demographic, socio-economic, dietary, examination, laboratory as well as questionnaire data administered by highly trained medical personnel [28], and it provides the data as public-use files. Every participant enrolled in NHANES gave written informed consent, and the procedures were sanctioned by the Institutional Review Board of the Centers for Disease Control and Prevention.

In total, 15882 individuals were identified from the 2011–2018 cycles of the NHANES (http://wwwn.cdc.gov/nchs/nhanes). In the process of data compilation, participants who lacked information on their CBC and blood manganese concentration were excluded. Additionally, the participants who had no data on demographics, body mass index (BMI), as well as alcohol use were excluded.

### Variables

In this investigation, the exposure variable was RDW. We divided manganese levels into three groups on the basis of their tertiles: low was to <8.08 ug/L (n = 5287); medium was≥8.08 ug/L to 10.75 ug/L (n = 5302), and high was >10.75 ug/L (n = 5293). Besides, the outcome variable, blood manganese concentration, was quantified by inductively coupled plasma-mass spectrometry(ICP-DRC-MS) technology. Manganese had a detection limit of 1.06 ug/L from 2011 to 2012 and 0.99 ug/L from 2013 to 2018. NHANES assigned samples with concentrations below the lower limit of detection (LLOD), a value of the detection limit divided by the square root of two. However, there were no participants who had blood manganese concentrations below the LLOD (0.99 ug/L and 1.06 ug/L). The following continuous variables were included: age, BMI(kg/m2), income(dollars/year), white blood cell(WBC) count, RBC count, hemoglobin, platelet count, Mean Platelet Volume(MPV), RDW, hematocrit, neutrophil, lymphocyte, monocyte, eosinophil and basophil counts. The categorical variables listed below were incorporated: gender, race, alcohol use, and educational level. Detailed information on all laboratory procedures is available at http://www.cdc.gov/nchs/about/major/nhanes/datalink.htm.

### Statistical analysis

The baseline characteristics are delineated based on the tertiles of blood manganese levels. Participant characteristics were displayed as means with standard deviations (SD) for continuous variables or as proportions for categorical variables. Differences in characteristics across manganese tertiles were examined utilizing one-way analysis of variance(ANOVA) (continuous variables) and chi-square test(categorical variables). In the first place, the correlation of blood manganese levels with RDW was analyzed in three distinct models employing a weighted multiple linear regression model. Model 1 represented an unadjusted variable model, while Model 2 was adjusted for age and gender. Model 3 incorporated covariates such as age, gender, BMI, race, income, educational level, and alcohol use. Manganese tertiles were also grouped for analysis. Weighted generalized linear models were adjusted for covariates and further stratified by age, gender, income, as well as educational level. We applied a two-piecewise regression model to determine whether blood manganese concentrations have a threshold effect on the RDW. A restricted cubic spline plot (RCS) was adopted to detect potential nonlinear relationships between blood manganese levels and the RDW, and the RCS models were further stratified by age, gender, income and educational level. To assess the robustness of the results, sensitivity analysis was performed following removing patients with iron deficiency. The data analyses were performed utilizing packages R(http://www.R-project.org). A *P*<0.05 was considered statistically significant.

## Results

### Baseline characteristics of participants

**Fig 1:**
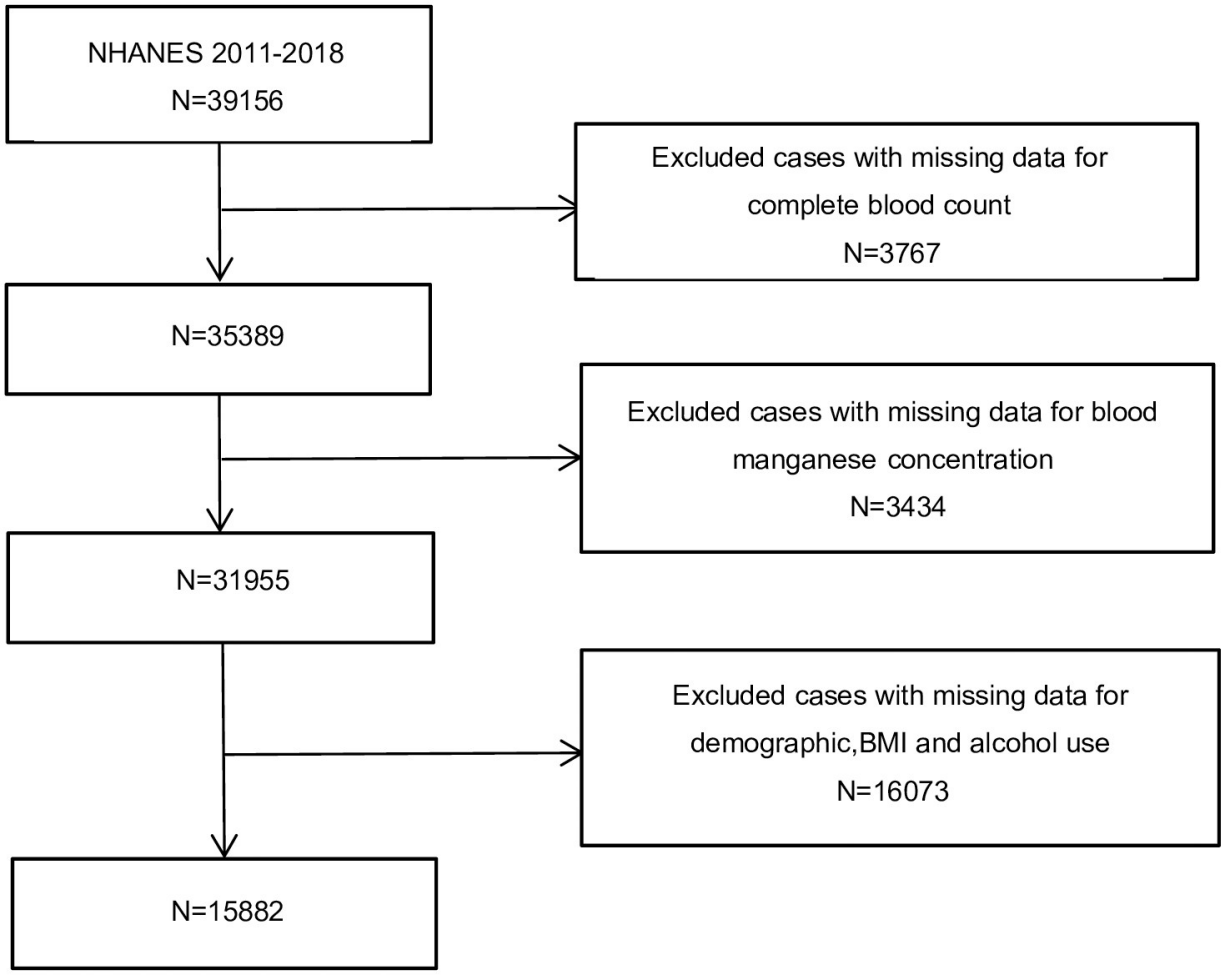
Flowchart of the sample selection from NHANES 2011–2018. A total of 39156 participants were enrolled at first, and after the exclusion of missing data for complete blood count (n = 3767), blood manganese concentration(n = 3434), demographic, BMI and alcohol use (n = 16073), 15882 eligible participants were included in our final analysis.

Data of a cross-sectional population of 15882 subjects was enrolled and the detailed process was shown in Fig 1. The average value of RDW at baseline was 13.7%±1.4%. The range of RDW was 10.8% to 29.8%. Blood manganese was categorized into three tertiles (Tertile 1: <8.08ug/L, Tertile 2: ≥8.08, ≤10.75ug/L, Tertile 3: >10.75ug/L). The baseline characteristics of the subjects based on the blood manganese levels are presented in Table 1. The results showed significant differences in all except the BMI, educational level, Mean Platelet Volume(fl), lymphocyte-, eosinophil-, and basophil counts. Age, gender, race, alcohol use, WBC, RBC, hemoglobin, platelet count, RDW, hematocrit, neutrophils and monocytes substantially differed between the manganese tertile groups (*P* < 0.001). Female gender was associated with higher blood manganese values. In addition, we observed that RDW was highest in the highest manganese tertile.

**Table 1:** Baseline characteristics of the participants according to tertiles of blood manganese levels in NHANES 2011–2018.

Manganese(ug/L)	Total	Tertile 1 (<8.08)	Tertile 2 (≥8.08,≤10.75)	Tertile3 (>10.75) N = 5293	*P*
	N = 15882	N = 5287	N = 5302		
Age (years)					<0.001
≤60	11071(69.7%)	3390 (64.1%)	3693 (69.7%)	3988 (75.3%)	
>60	4811(30.3%)	1897 (35.9%)	1609 (30.3%)	1305 (24.7%)	
Gender					<0.001
Male	7732(48.7%)	3112 (58.9%)	2665 (50.3%)	1955 (36.9%)	
Female	8150(51.3%)	2175 (41.1%)	2637 (49.7%)	3338 (63.1%)	
BMI (Kg/m2)					0.066
<18.5	296(1.9%)	103 (1.9%)	86 (1.6%)	107 (2%)	
18.5≥,≤24.9	4447(28.0%)	1438 (27.2%)	1464 (27.6%)	1545 (29.2%)	
>25	11139(70.1%)	3746 (70.9%)	3752 (70.8%)	3641 (68.8%)	
Race					<0.001
Mexican American	2121(13.4%)	471 (8.9%)	730 (13.8%)	920 (17.4%)	
Other Race	4349(27.4%)	868 (16.4%)	1370 (25.8%)	2111 (39.9%)	
Non-Hispanic White	5795(36.5%)	2116 (40%)	2093 (39.5%)	1586 (30%)	
Non-Hispanic Black	3617(22.8%)	1832 (34.7%)	1109 (20.9%)	676 (12.8%)	
Income(dollars/year)					0.162
<20000	4468(28.1%)	1526 (28.9%)	1501 (28.3%)	1441 (27.2%)	
≥20000	11414(71.9%)	3761 (71.1%)	3801 (71.7%)	3852 (72.8%)	
Educational level					0.566
<High school	7433(46.8%)	2504 (47.4%)	2457 (46.3%)	2472 (46.7%)	
≥High school	8449(53.2%)	2783 (52.6%)	2845 (53.7%)	2821 (53.3%)	
Alcohol use					<0.001
Yes	6913(43.5%)	2515 (47.6%)	2358 (44.5%)	2040 (38.5%)	
No	8969(56.5%)	2772 (52.4%)	2944 (55.5%)	3253 (61.5%)	
WBC(*10^9^/L)	7.2 ± 2.3	7.1 ± 2.4	7.2 ± 2.3	7.3 ± 2.3	<0.001
RBC(*10^12^/L)	4.7 ± 0.5	4.6 ± 0.5	4.7 ± 0.5	4.7 ± 0.5	<0.001
Hemoglobin(g/dL)	14.0 ± 1.5	13.9 ± 1.4	14.1 ± 1.4	13.8 ± 1.7	<0.001
Platelet count(*10^9^/L)	239.3 ± 62.1	232.6 ± 59.4	238.9 ± 59.9	246.5 ± 66.0	<0.001
MPV(fl)	8.4 ± 0.9	8.4 ± 0.9	8.4 ± 0.9	8.3 ± 0.9	0.064
RDW(%)	13.7 ± 1.4	13.4 ± 1.1	13.4 ± 1.1	13.8 ± 1.8	<0.001
Hematocrit(%)	41.4 ± 4.2	41.3 ± 4.0	41.8 ± 4.0	41.0 ± 4.6	<0.001
Neutrophil count(*10^9^/L)	4.2 ± 1.7	4.2 ± 1.7	4.2 ± 1.7	4.3 ± 1.7	<0.001
Lymphocyte count(*10^9^/L)	2.1 ± 1.1	2.1 ± 1.3	2.2 ± 0.9	2.2 ± 1.0	0.229
Monocyte count(*10^9^/L)	0.6 ± 0.2	0.6 ± 0.2	0.6 ± 0.2	0.5 ± 0.2	<0.001
Eosinophil count(*10^9^/L)	0.2 ± 0.2	0.2 ± 0.2	0.2 ± 0.2	0.2 ± 0.2	0.636
Basophil count(*10^9^/L)	0.0 ± 0.1	0.0 ± 0.1	0.0 ± 0.1	0.0 ± 0.1	0.983

Abbreviations:BMI, body mass index; WBC, white blood cell; RBC, red blood cell; MPV, Mean platelet volume; RDW, red blood cell distribution width;

Note: Continuous variables are presented as weighted means (standard error), while categorical variables are presented as weighted population (percentage), unless otherwise specified. Group differences based on manganese levels were evaluated using the one-way ANOVA test for continuous variables and the chi-square test for categorical variables.

### Associations between blood manganese levels and RDW

Table 2 indicates the association between blood manganese levels and RDW. A positive association between blood manganese levels and RDW was detected in model 1 (β = 0.079, 95%CI: 0.073–0.084, *P*<0.001), model 2 (β = 0.075, 95%CI: 0.069–0.080, *P*<0.001), and model 3 (β = 0.100, 95%CI: 0.095–0.106, *P*<0.001). Nevertheless, this positive correlation disappeared following the conversion of blood manganese to tertiles. For cases with the lowest tertile of blood manganese levels, there was a significant negative association between blood manganese levels and RDW in model 1 (β = -0.138, 95%CI: -0.165–-0.111, *P*<0.001), model 2 (β = -0.140, 95%CI: -0.167–0.113, *P*<0.001), and model 3 (β = -0.081, 95%CI: -0.107–-0.055, *P*<0.001). For participants with the middle tertile of blood manganese levels, a positive association existed with blood manganese levels and RDW in model 1 (β = 0.058, 95%CI: 0.020–0.096, *P*<0.001), model 2 (β = 0.054, 95%CI: 0.016–0.092, *P*<0.001), and model 3 (β = 0.072, 95%CI: 0.037–0.101, *P*<0.001). Moreover, the positive trend persisted in participants with the highest tertile of blood manganese levels in model 1 (β = 0.138, 95%CI: 0.126–0.150, *P*<0.001), model 2 (β = 0.132, 95%CI: 0.120–0.144, *P*<0.001), and model 3 (β = 0.139, 95%CI: 0.127–0.150, *P*<0.001).

**Table 2:** Association between blood manganese levels and RDW among United States adults in NHANES 2011–2018.

Manganese(tertiles)(ug/L)	Model1:β(95%CI)	Model2:β(95%CI)	Model3:β(95%CI)
	*P*	*P*	*P*
Total	0.079(0.073–0.084) <0.001	0.075(0.069–0.080) <0.001	0.100(0.095–0.106) <0.001
<8.08	-0.138(-0.165–0.111) <0.001	-0.140(-0.167–0.113) <0.001	-0.081(-0.107–0.055) <0.001
≥8.08,≤10.75	0.058(0.020–0.096) <0.001	0.054(0.016–0.092) <0.001	0.072(0.037–0.101) <0.001
>10.75	0.138(0.126–0.150) <0.001	0.132(0.120–0.144) <0.001	0.139(0.127–0.150) <0.001

Note:

Model 1: No covariates were adjusted.

Model 2: Age and gender were adjusted.

Model 3: Age, gender, BMI, race, income, educational level and alcohol use were adjusted.

CI, confidence interval.

To determine whether the correlation between blood manganese levels and RDW was consistent across population settings, subgroup analysis was performed (Table 3). Consistent results were observed when analyses were stratified by age, gender, income and educational level. Finally we found that the association between blood manganese levels and RDW was strongest in women.

**Table 3:** Subgroup analysis of association between blood manganese levels and RDW among United States adults in NHANES 2011–2018.

	Model1:β(95%CI)	Model2:β(95%CI)	Model3:β(95%CI)
	*P*	*P*	*P*
Age(years)			
≤60	0.102(0.096–0.108) <0.001	0.094(0.088–0.100) <0.001	0.112(0.106–0.118) <0.001
>60	0.038(0.028–0.048) <0.001	0.040(0.030–0.050) <0.001	0.056(0.045–0.066) <0.001
Gender			
Males	0.018(0.010–0.026)	0.024(0.017–0.031)	0.039(0.031–0.047)
	<0.001	<0.001	<0.001
Females	0.107(0.100–0.115)	0.115(0.108–0.123)	0.133(0.126–0.141)
	<0.001	<0.001	<0.001
Income (dollars/year)			
<20000	0.087(0.076–0.098)	0.090(0.079–0.100)	0.109(0.099–0.120)
	<0.001	<0.001	<0.001
≥20000	0.076(0.070–0.082)	0.081(0.075–0.087)	0.097(0.091–0.103)
	<0.001	<0.001	<0.001
Educational level			
<High school	0.087(0.080–0.096)	0.093(0.085–0.100)	0.111(0.103–0.119)
	<0.001	<0.001	<0.001
≥High school	0.071(0.063–0.078)	0.074(0.066–0.081)	0.090(0.083–0.098)
	<0.001	<0.001	<0.001

Note:

Model 1: No covariates were adjusted.

Model 2: Age and gender were adjusted.

Model 3: Age, gender, BMI, race, income, educational level as well as alcohol use were adjusted.

In the subgroup analysis stratified by age, gender, income and educational level, the model is not adjusted for the stratification variable itself.

CI, confidence interval.

**Fig 2:**
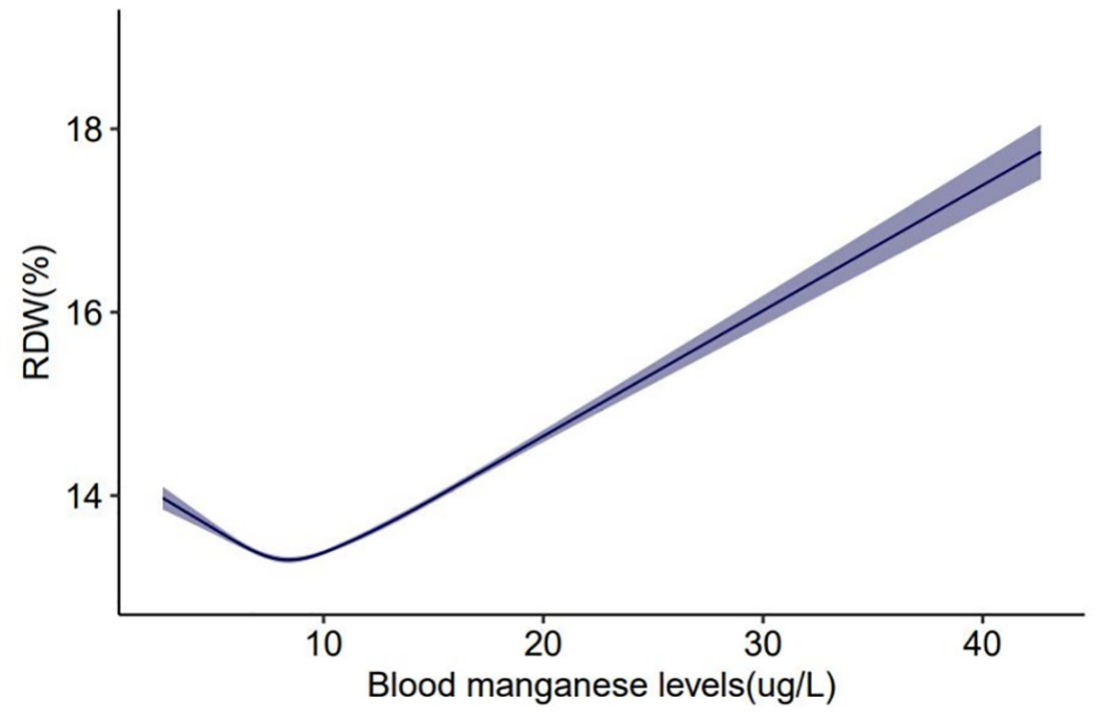
The association between blood manganese levels and RDW in all participants. Solid line represents the smooth curve fit between variables. Shadow represents the 95% of confidence interval from the fit.

**Fig 3:**
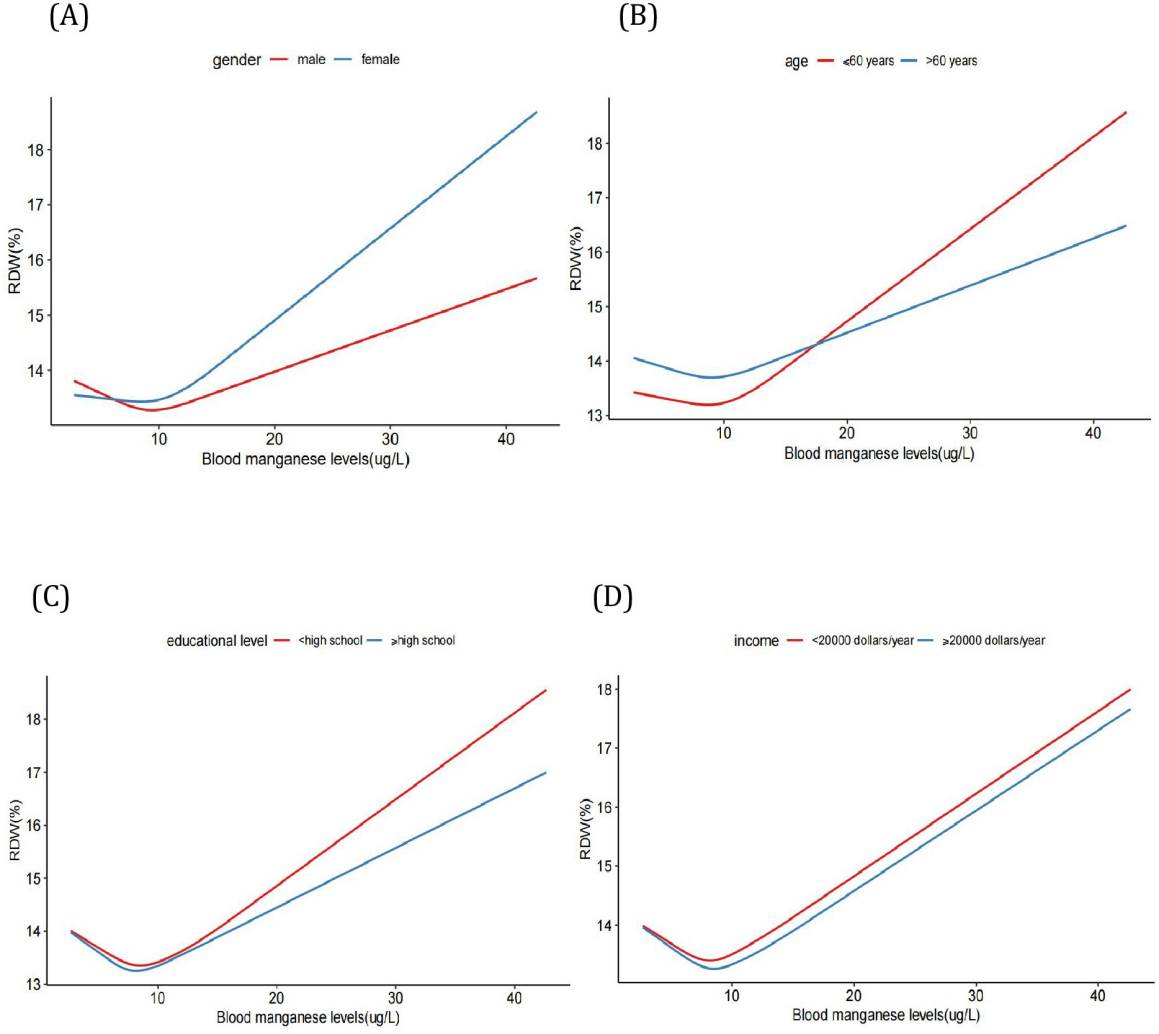
The association between blood manganese levels and RDW stratified by gender, age, educational level and income. (A) Model is stratified by gender. (B) Model is stratified by age. (C) Model is stratified by educational level. (D) Model is stratified by income. Age, gender, race, income, educational level and alcohol are adjusted in all modes. The model is not adjusted for the stratification variable itself.

Furthermore, we employed the smooth curve fittings to characterize the association between blood manganese levels and RDW, which are shown in Figs 2 and 3. The smooth curve suggested that the J-shaped relationship between blood manganese levels and RDW was maintained in age, gender, income and educational level groups after adjusting for age, gender, race, income, educational level and alcohol use(*P*<0.001). By the two-piecewise linear regression model, we calculated that the inflection point was 7.32(ug/L) in the general population following adjusting for age, gender, race, income, educational level, as well as alcohol use (Table 4). Furthermore, the log-likelihood ratio test showed that the value of *P* was <0.001, which verified the difference between a two-piece linear regression model and a linear regression model. In a two-piece linear regression model, the RDW values exhibited a significant decrease with the increase in blood manganese (per ug/L: (β = -0.144, 95%CI:0.180–0.107, *P*<0.001,Table 4)) in participants on the left of the inflection point (<7.32 ug/L). Conversely, the analysis illustrated that the values of RDW were significantly positively correlated with higher blood manganese levels(per ug/L: (β = 0.123,95%CI:0.117–0.130, *P*<0.001, Table 4)) in participants on the right of the inflection point (≥7.32 ug/L). In the sensitivity analysis, we excluded 676 patients with iron deficiency, and the association between the blood manganese levels and RDW remained reliable (Table 5).

**Table 4:** Threshold effect analysis of blood manganese levels on RDW using the two-piecewise linear regression model.

Blood manganese	Adjusted β(95%CI)	*P*
Fitting by the standard linear model	0.079(0.073–0.084)	<0.001
Fitting by the two-piecewise linear model		
Inflection point		
Blood manganese <7.32 (ug/L)	-0.144(-0.180–0.107)	<0.001
Blood manganese≥7.32 (ug/L)	0.123(0.117–0.130)	<0.001
Log-likelihood ratio test	<0.001	

Note: Age, gender, BMI, race, income, educational level and alcohol use were adjusted.

CI, confidence interval.

**Table 5:** Association between blood manganese levels and RDW among United States adults in NHANES 2011–2018. (sensitivity analysis).

Manganese(tertiles)(ug/L)	Model1:β(95%CI)	Model2:β(95%CI)	Model3:β(95%CI)
	*P*	*P*	*P*
Total	0.067(0.062–0.072)	0.071(0.066–0.077)	0.086(0.081–0.091)
	<0.001	<0.001	<0.001
<8.08	-0.131(-0.158–0.105)	-0.104(-0.130–0.079)	-0.081(-0.106–0.056)
	<0.001	<0.001	<0.001
≥8.08,≤10.75	0.051(0.014–0.088)	0.059(0.023–0.095)	0.060(0.026–0.094)
	<0.001	<0.001	<0.001
>10.75	0.122(0.111–0.133)	0.120(0.109–0.131)	0.124(0.114–0.135)
	<0.001	<0.001	<0.001

Note:

Model 1: No covariates were adjusted.

Model 2: Age and gender were adjusted.

Model 3: Age, gender, BMI, race, income, educational level and alcohol use were adjusted.

CI, confidence interval.

## Discussion

This study sought to investigate independent correlations between blood manganese levels and RDW among participants. The study revealed two key findings. Firstly, a J-shaped relationship between blood manganese levels and RDW was identified in participants, consistently observed across various population settings after adjusting for age, gender, BMI, race, income, educational level, and alcohol use. The threshold effect analysis revealed the turning point of blood manganese(7.32 ug/L)in the general population. Secondly, the positive correlation between RDW and blood manganese levels was more pronounced among females where blood manganese was greater than the inflection point(≥7.32 ug/L).

Individuals encounter manganese through various environmental channels, predominantly via oral, inhalation, dermal, and intravenous means [29]. Nevertheless, for the broader populace, the principal non-occupational avenue of manganese exposure is ingestion, given that both food and potable water encompass manganese [30]. As a cofactor of manganese superoxide dismutase, manganese contributes significantly to cellular metabolism [31]. Manganese supplementation in moderation may prevent oxidative stress and promote the development of bones and skeletal muscles [32]. One of the essential components of bones is manganese [33]. As indicated by research finding, about 40% of the body’s manganese content is attributed to the presence of manganese in bones, establishing bones as the primary repository for this element [34]. In a mouse study, the half-life for manganese was found to be 143 days, in contrast to 8.5 years in human bones [35]. Consequently, the impact of excessive manganese exposure on physical health is sustained and profound.

The link between blood manganese levels and RDW is not supported by substantial direct evidence. The RDW is a simple parameter that reflects the heterogeneity of circulating erythrocyte volume, and an increase in RDW characterizes anemia. Patients with iron deficiency anemia typically have higher RDW. Nevertheless, iron-deficient patients excessively exposed to manganese frequently run the risk of iron deficiency exacerbation, leading to an increase in RDW [36]. Approximately 50 years ago, a cohort study conducted by Mena et al. proposed that anemic patients exhibited a six-fold increase in red cell manganese concentration [37]. Since then, a few studies have demonstrated that iron deficiency was associated with increased blood manganese concentration in the general population due to the fact that manganism can interfere with the transport mechanism of iron [38–40]. High levels of blood manganese were found to be independently associated with a higher level of hemoglobin in patients undergoing maintenance hemodialysis, according to another cohort study [41].

The mechanisms underlying the cross-sectional association of blood manganese levels with RDW are not fully understood and could only be speculated. Firstly, iron deficiency can lead to an increase in RDW [42]. Manganese has the potential to modulate transferrin protein, influencing iron absorption and expediting the transport of serum iron to the brain [43, 44]. Secondly, the hypothetical mechanisms of manganese toxicity center on oxidative stress and inflammation, resulting in mitochondrial dysfunction and dysregulated autophagy [45–47]. Red blood cell production or destruction may contribute to an increase in RDW [48]. Oxidative stress and inflammation can regulate the generation and apoptosis of red blood cells by affecting bone marrow function and iron metabolism, thus increasing RDW [49]. This phenomenon is common in hemolysis [50]. Thirdly, Chronic kidney disease (CKD) is recognized as a risk factor for anemia, typically usually related to erythropoietin(EPO) deficiency [51]. Manganese exposure may potentially lead to renal system damage and impairment of EPO synthesis, consequently interfering with the maturation of blood progenitor cells [52–54], which may potentially contribute to elevated RDW.

Utilizing a nationally representative sample from the United States and incorporating data that had been consolidated for nearly a decade constitutes the study’s greatest strength. This study is the first to examine the correlation between blood manganese levels and RDW in the general population. Nevertheless, our study had some limitations that deserve discussion. In the first place, the cross-sectional nature of this study confined the results to a correlation rather than causality. The precise mechanism by which blood manganese levels and RDW interact among adults in the United States remains unknown. Second, blood manganese levels alone may not reflect manganese metabolism as well as distribution in these participants, as manganese concentrations in the bone, or hair were not examined. Finally, reliance on data from an American population limited the generalizability of our findings. Consequently, it is expected that fundamental mechanistic research and large sample prospective studies will be conducted to further determine the relationship blood manganese levels and RDW.

## Conclusions

We determined a J-shaped relationship between blood manganese levels and RDW with an inflection point at 7.32(ug/L). Despite the fact that further work should be done to examine these relationships, our study emphasizes the need to address environmental exposures to manganese in the general population and in particularly affected groups.
